# Congenital Hypothyroidism and Brain Development: Association With Other Psychiatric Disorders

**DOI:** 10.3389/fnins.2021.772382

**Published:** 2021-12-09

**Authors:** Katsuya Uchida, Mao Suzuki

**Affiliations:** ^1^Laboratory of Information Biology, Graduate School of Information Sciences, Tohoku University, Sendai, Japan; ^2^Laboratory of Biomodeling, Graduate School of Information Sciences, Tohoku University, Sendai, Japan

**Keywords:** thyroid hormone, hypothyroid, developmental disorder, parvalbumin, psychiatric disorder, MeCP2

## Abstract

Thyroid hormones play an important role in brain development, and thyroid hormone insufficiency during the perinatal period results in severe developmental delays. Perinatal thyroid hormone deficiency is clinically known as congenital hypothyroidism, which is caused by dysgenesis of the thyroid gland or low iodine intake. If the disorder is not diagnosed or not treated early, the neuronal architecture is perturbed by thyroid hormone insufficiency, and neuropathological findings, such as abnormal synapse formation, defects in neuronal migration, and impairment of myelination, are observed in the brains of such patients. Furthermore, the expression of psychiatric disorder-related molecules, especially parvalbumin, is significantly decreased by thyroid hormone insufficiency during the perinatal period. Animal experiments using hypothyroidism models display decreased parvalbumin expression and abnormal brain architecture, and these experimental results show reproducibility and stability. These basic studies reinforce the results of epidemiological studies, suggesting the relevance of thyroid dysfunction in psychiatric disorders. In this review, we discuss the disruption of brain function associated with congenital hypothyroidism from the perspective of basic and clinical research.

## Introduction

Thyroid hormones are synthesized in and released by the thyroid gland, with thyroxine (T4) comprising the highest concentration of these hormones. T4 is released from the thyroid gland and converted to triiodothyronine (T3) by deiodinase; T3 is highly biologically active as a transcription factor that plays important roles in brain development. This includes its roles in glial myelination, neuronal migration, cortical layer formation, synaptogenesis, and neurogenesis (Nicholson and Altman, 1972; Oppenheimer and Schwartz, 1997; Koibuchi and Chin, 2000; Uchida et al., 2005). Therefore, thyroid hormones during the perinatal period are important for normal development of the brain, and congenital hypothyroidism causes serious developmental delay if proper treatment is not implemented immediately after birth in such patients (Morreale de Escobar et al., 1987; Rastogi and LaFranchi, 2010). Thyroid dysfunction can be clinically detected by mass screening immediately after birth, and developmental disorders can be avoided by treatment with levothyroxine (T4).

Therapy for thyroid dysfunction has been established; however, neuroscientific research using rodent models is still ongoing because thyroid hormones are involved in diverse aspects of neurodevelopment, and thyroid hormone research in turn has the potential to provide new insights. A typical neuropathological finding caused by thyroid hormone insufficiency is a decrease in the parvalbumin of GABAergic neurons. This phenomenon is of interest to many researchers because it is observed not only in thyroid hormone insufficiency, but also in the dysfunction of thyroid hormone receptors and iodothyronine deiodinase (Berbel et al., 1996; Gilbert et al., 2007; Wallis et al., 2008; Barez-Lopez et al., 2019). In other words, the decrease in parvalbumin in GABAergic neurons is closely linked to the thyroid system. In addition, in recent years, a decrease in parvalbumin has been observed in the postmortem brains of patients with autism and schizophrenia, reaffirming the importance of thyroid hormone research in these conditions (Hashimoto et al., 2003; Lawrence et al., 2010; Soghomonian et al., 2017). Furthermore, parvalbumin neurons are lost in the cerebral cortex of mice lacking methyl CpG binding protein 2 (MeCP2), the gene responsible for Rett syndrome (RTT) (Fukuda et al., 2005). Recently, abnormalities in thyroid function in RTT patients have also been reported (Stagi et al., 2015). Herein, we present the neuropathological findings observed in experimental hypothyroidism (animal study) and congenital hypothyroidism, discuss the relationship between some psychiatric disorders and hypothyroidism, and review the importance of thyroid hormones in brain development.

## Role of Thyroid Hormone on the Brain Architecture

The role of thyroid hormones in brain development has long been studied, and numerous studies have been published to date. Congenital hypothyroidism that is not diagnosed or not treated early causes developmental delay, which, as mentioned above, is triggered by abnormalities in neural architecture during brain development. Notably, maternal hypothyroidism during pregnancy has long-lasting effects on the cortical morphology of their offspring, with specific effects reflecting both the severity and timing of maternal thyroid hormone insufficiency (Lischinsky et al., 2016). Furthermore, Cooper et al. (2019) reported that individuals with severe congenital hypothyroidism are at risk of developing white matter microstructural abnormalities, despite early detection and treatment. In congenital hypothyroidism, neuropathological features such as abnormalities in neuritogenesis of Purkinje cells in the cerebellum, myelin sheath hypoplasia in myelinated nerves, and dysgenesis of dendrite spine formation are observed in the developmental and mature brain. However, the effects of thyroid hormone deficiency are not only observed in local cellular structures, but also in neural architecture and signal transmission between the cerebral hemisphere *via* the corpus callosum (Berbel et al., 1993; Samadi et al., 2015). Although cell positioning during corticogenesis follows an inside-out pattern, radial neurogenic gradients are more diffuse than in normal animals. This difference in radial migration may be attributed to reduced reelin mRNA and protein in Cajal–Retzius cells observed in hypothyroid animals during the perinatal period. Since the administration of T3 to hypothyroid rats restores reelin mRNA expression both *in vitro* and *in vivo* (Alvarez-Dolado et al., 1999), the formation of radial neurogenic gradients may be highly dependent on thyroid hormones. Regarding the commissural fibers, the commissural neurons that form cortical layer II/III in the cortex are connected to the contralateral hemisphere *via* the corpus callosum. In general, retrograde neural tracers administered to the primary auditory cortex are widely distributed in the cortical layers of the contralateral side; however, in hypothyroid animals, tracer signals converge in cortical layers IV–V of the contralateral side of the primary auditory cortex (Berbel et al., 1993). Furthermore, Goodman and Gilbert (2007) reported that thyroid hormone insufficiency induced cellular malformation in the corpus callosum. Abnormalities in neural connections between the cerebral hemispheres, hence, would have a very strong impact on integrated brain functions (Table 1). In fact, abnormalities associated with commissural fibers have been observed not only in hypothyroidism but also in autism spectrum disorder (ASD) and attention deficit hyperactivity disorder (Casanova et al., 2011; Qiu et al., 2011; Ameis et al., 2016), indicating that defects in brain structures involved in functional integration affect behavioral expression. Thus, thyroid hormones participate in various aspects of the developing brain.

**TABLE 1:** Role of thyroid hormone on the brain architecture.

Species	State	References	Histological features
Human	Hypothyroidism	Lischinsky et al. (2016)	Cortical thinning and thickening
Human	Hypothyroidism	Cooper et al. (2019)	White matter microstructural abnormalities
Rat	Experimental hypothyroidism (MMI)	Berbel et al. (1993)	Dysgenesis of cortical layers and callosal connections
Human	Hypothyroidism	Samadi et al. (2015)	Dysgenesis of the corpus callosum
Rat	Experimental hypothyroidism (MMI)	Alvarez-Dolado et al. (1999)	Decreased in reelinRNA and protein
Rat	Experimental hypothyroidism (PTU)	Goodman and Gilbert (2007)	Cellular malformation in the corpus callosum

*MMI, methimazole; PTU, propylthiouracil.*

## Involvement of Thyroid Hormone on Parvalbumin Expression

Parvalbumin is a calcium-binding protein and a type of albumin with a small molecular weight (12 kD) (Arif, 2009). Parvalbumin is expressed in GABAergic neurons in the central nervous system, and parvalbumin-expressing neurons are mainly observed in the cortex, hippocampus, cerebellum, and reticular hypothalamic nucleus (Celio, 1986, 1990; Kosaka et al., 1987). Parvalbumin-expressing GABAergic neurons are generated from the medial ganglionic eminence and migrate tangentially to their respective destination areas (Danglot et al., 2006). In the cortex and hippocampus, the developmental expression of parvalbumin mRNA is observed from approximately postnatal day 10, and gradually increases daily (de Lecea et al., 1995). Parvalbumin neurons differentiate into basket and chandelier cells and function as fast-spiking interneurons (Hu et al., 2014).

Berbel et al. (1996) first reported the microstructural differences in parvalbumin neurons in adult hypothyroid rats. This report indicated that hypothyroid rats showed dysgenesis of parvalbumin-positive terminal puncta in the neocortex, while there were no differences observed in the number of parvalbumin neurons. Guadano-Ferraz et al. (2003) also reported that thyroid hormone receptor alpha 1-deficient mice displayed a decrease in the density of parvalbumin-positive terminals in the hippocampus. These two reports mainly showed the effect of hypothyroidism on the nerve endings of parvalbumin neurons. In contrast, a decrease in the number of parvalbumin neurons in the cortex and other regions has been shown in monocarboxylate transporter 8 (MCT8) and deiodinase type 2 (Dio2)-double deficient mice (Barez-Lopez et al., 2019). MCT8 actively transports a variety of iodothyronines, including thyroid hormones (T3 and T4) (Friesema et al., 2003), and Dio2 activates thyroid hormones by converting the prohormone T4 to bioactive T3 (Croteau et al., 1996). In mice, single mutations of MCT8 or Dio2 do not cause a significant decrease in the number of parvalbumin neurons due to compensatory effects. Therefore, double deficiency of MCT8 and Dio2 as well as severe thyroid hormone dysfunction may lead to a decrease in the number of parvalbumin neurons in mice. Gilbert et al. (2007) reported in detail the effects of thyroid hormone insufficiency on parvalbumin expression. Interestingly, animal models of congenital hypothyroidism display a significant decrease in the number of parvalbumin neurons during the juvenile phase, and the levels of parvalbumin expression slightly catch up with those of normal animals, with recovery of serum thyroid hormone levels after the termination of treatment with antithyroid agents. However, the degree of the decrease in parvalbumin neuron number and its subsequent recovery is dependent on the concentration of antithyroid agents, and significant recovery of the number of parvalbumin-expressing neurons was observed in the low concentration exposure group, but not in the high concentration exposure group. Thus, even though the levels of serum thyroid hormone completely recover in adulthood, animals that experience thyroid hormone deficiency during the perinatal period still retain the signatures of temporary hormonal defects in parvalbumin neurons in the adult brain. As a result of this occurrence, dysfunction of neuron-specific K(+)/Cl(−) co-transporter (KCC2) and a delayed onset of synaptic inhibition have been observed accordingly (Friauf et al., 2008; Yi et al., 2014). In general, during the first 2 weeks after birth, synaptic transmission *via* the inhibitory transmitter changes from excitatory depolarizing to inhibitory hyperpolarizing effects in GABAergic neurons (Cherubini et al., 1991; Rivera et al., 1999). Therefore, delay in the functional conversion of inhibitory neurons may significantly perturb the integrative function of inhibitory neural circuits in the hypothyroid brain.

On the other hand, just as treatment with levothyroxine avoids developmental delay accompanying congenital hypothyroidism in humans, thyroid hormone replacement immediately after birth can prevent a decrease in the number of parvalbumin neurons in rodents (Gilbert et al., 2007; Uchida et al., 2014, 2021). Thyroid hormone replacement after postnatal day 14 has no effect on the number of parvalbumin neurons, suggesting that a critical period of thyroid hormone sensitivity exists before this day (Gilbert et al., 2007; Uchida et al., 2014, 2021). Interestingly, a transient increase in blood thyroid hormone levels was observed around postnatal day 14 (Fishman et al., 1982; Calikoglu et al., 1996; Hadj-Sahraoui et al., 2000). Hence, the hormonal surge and/or the abundance of hormones in the early postnatal period might be important for normal neurodevelopment, including maturation of parvalbumin neurons. As mentioned above, parvalbumin expression and its morphogenesis have been observed to correlate with thyroid hormone levels; however, the direct or indirect action of TH on the transcription of PV genes remains unclear.

Similar to observations in patients with MCT8 mutations, loss of parvalbumin expression has been observed in the brains of patients with schizophrenia and autism (Hashimoto et al., 2003; Lopez-Espindola et al., 2014; Filice et al., 2020). Since these psychiatric disorders are observed in perturbation of executive functions, parvalbumin expression in the human prefrontal cortex has been preferentially analyzed accordingly. Although there are differences in the results among studies, a decrease in parvalbumin expression in the prefrontal cortex has been confirmed in schizophrenia and autism (Beasley and Reynolds, 1997; Hashemi et al., 2017; Ariza et al., 2018; Kaar et al., 2019) (Table 2). Therefore, a parvalbumin hypothesis for developmental delay has been proposed (Filice et al., 2020).

**TABLE 2:** Morphological abnormalities of parvalbumin neurons in the brain.

Species	State	References	Histological features
Rat	Experimental hypothyroidism (MMI)	Berbel et al. (1996)	The density of nerve terminal ↓
Mouse	Thyroid hormone receptor alpha1 deficient	Guadano-Ferraz et al. (2003)	The density of nerve terminal ↓
Mouse	Mct8/Dio2 double KO	Barez-Lopez et al. (2019)	The number of PV neurons (signals)↓
Rat	Experimental hypothyroidism (PTU)	Gilbert et al. (2007)	The number of PV neurons (signals)↓
Mouse	Experimental hypothyroidism (MMI/perchlorate)	Uchida et al. (2021)	The number of PV neurons (signals)↓
Human	Mct8 mutation	Lopez-Espindola et al. (2014)	The number of PV neurons (signals)↓
Human	Schizophrenia	Hashemi et al. (2017)	The number of PV neurons (signals)↓
Human	Autism	Filice et al. (2020)	The number of PV neurons (signals)↓
Human	Schizophrenia	Beasley and Reynolds (1997)	The number of PV neurons (signals)↓
Human	Autism	Ariza et al. (2018)	The number of PV neurons (signals)↓
Human	Schizophrenia	Kaar et al. (2019)	The number of PV neurons (signals)↓

*MMI, methimazole; PTU, propylthiouracil.*

## Relationship Between Thyroid Disease and Psychiatric Disorders (Schizophrenia and Autism)

An increased prevalence of thyroid disorders has been noted in families of individuals with schizophrenia (DeLisi et al., 2000; Palha and Goodman, 2006; Radhakrishnan et al., 2013; Gyllenberg et al., 2016; Sharif et al., 2018). Radhakrishnan et al. (2013) reported that in a retrospective hospital-based study, hypothyroidism was observed in 25% of patients with schizophrenia. Sharif et al. (2018) also reported that the population of patients with schizophrenia in hypothyroid patients was higher than that in controls. Generally, the prevalence of schizophrenia is approximately 1%, but there is a twofold increase in the incidence rate of schizophrenia in patients with hypothyroidism. These large-scale studies show a higher interaction between hypothyroidism and schizophrenia, although the functional relevance of these disorders remains unclear. Since antipsychotics affect thyroid hormone secretion and conversion of T4 toT3 (Terao et al., 1995; Langlois et al., 2001), it should also be considered whether medication affects thyroid status, so as to estimate the relationship between thyroid state and schizophrenia more accurately. According to Melamed et al. (2020), the increased rate of hypothyroidism in patients with schizophrenia after, but not before, the diagnosis of schizophrenia suggests that antipsychotic medications may affect thyroid hormone levels. However, the principal preoccupation is whether perinatal thyroid hormone deficiency is associated with the onset of schizophrenia. According to Gyllenberg et al. (2016), maternal hypothyroxinemia may be associated with an increased risk for the onset of schizophrenia, suggesting an association between low maternal thyroxine and increased odds of offspring schizophrenia. Therefore, congenital hypothyroidism (maternal hypothyroidism) is a potential risk factor for the onset of schizophrenia.

It has been reported that maternal hypothyroidism is associated with an increased risk of ASD (Roman et al., 2013; Chang and Shin, 2014; Getahun et al., 2018; Ge et al., 2020) and that hypothyroid animal models are useful for understanding the molecular mechanisms of ASD (Sadamatsu et al., 2006; Berbel et al., 2014). In a population-based study, there were no strong associations between neonatal thyroid hormones and ASD, but subgroups of newborns with the lowest T4 levels exhibited modestly increased ASD risk (Lyall et al., 2017). Although no significant differences were reported in the levels of serum T4, T3, and thyroid stimulating hormone (TSH) in patients with ASD compared to reference samples (Cohen et al., 1980), this study analyzed thyroid function in children aged 10–12 years with ASD in comparison to normal children. Since mild maternal thyroid hormone insufficiency during the perinatal period affects brain formation in fetuses and neonatal infants, measurement of postnatal hormone levels may provide a clue to its relevance to ASD. In fact, Lischinsky et al. (2016) reported that even mild variations in maternal thyroid hormones permanently affect the offspring cortex. Furthermore, although early treatment of congenital hypothyroidism prevents developmental delay, affected children still exhibit subtle persistent neurocognitive deficits, such as poor attention (Rovet and Hepworth, 2001). In an overall evaluation of offspring, there may be a rigid functional correlation between maternal hypothyroidism during the perinatal period and ASD in offspring (Table 3). In subsequent studies, single nucleotide polymorphisms in the ligand-binding domain of thyroid hormone receptors were found in patients with ASD (Kalikiri et al., 2017). Furthermore, alterations in thyroid hormone-dependent genes have been observed in the postmortem brains of humans with ASD (Khan et al., 2014). Hence, the risk of developing autism may be associated not only with an underactive the thyroid gland, but also with a defect in the process of hormone functioning or in the rate of transcribed products. More clinical studies and basic research using animal models are needed to better understand the detailed functional relationship between thyroid diseases and ASD.

**TABLE 3:** Relationship between thyroid diseases and psychiatric disorders.

Species	State	References	Relationship
Human	Schizophrenia	DeLisi et al. (2000)	Increased prevalence of thyroid function disorders
Human	Schizophrenia	Radhakrishnan et al. (2013)	Increased prevalence of thyroid function disorders
Human	Schizophrenia	Gyllenberg et al. (2016)	Maternal hypothyroxinemia
Human	Hypothyroidism	Sharif et al. (2018)	Risk factor for schizophrenia
Human	Maternal hypothyroxinemia	Roman et al. (2013)	Risk factor for ASD
Human	Maternal hypothyroxinemia	Chang and Shin (2014)	Risk factor for ASD
Human	Maternal hypothyroxinemia	Getahun et al. (2018)	Risk factor for ASD
Human	Maternal hypothyroxinemia	Ge et al. (2020)	Risk factor for ASD
Human	Autism	Cohen et al. (1980)	No correlation of thyroid function disorders
Human	Newborns with low T4 levels	Lyall et al. (2017)	Risk factor for ASD
Human	Hypothyroidism	Rovet and Hepworth (2001)	Onset of attention deficit

*ASD, Autism spectrum disorder.*

## Thyroid Function Disorders and Rett Syndrome

The relationship between thyroid function disorders and RTT is unclear. RTT is a rare genetic disorder caused by mutations or deletions in a gene called MeCP2 on the X chromosome, resulting in severe mental and physical disabilities (Braddock et al., 1993; Gold et al., 2018). RTT is also rarely caused by abnormalities in the CDKL5 and FOXG1 genes (Guerrini and Parrini, 2012). Human autopsy brain tissue from with patients with RTT displays decreased in dendritic spines and neurotrophic factors (Belichenko and Dahlstrom, 1995; Lipani et al., 2000), and such histological impairment may be a leading cause of developmental delay. Partially common neuropathological findings are observed in both RTT and congenital hypothyroidism (Eayrs, 1960; Armstrong, 1997); this is of interest as a research subject for basic medical researchers. Currently, there are less than 10 publications on the relationship between RTT and thyroid state, and several papers have reported very interesting results. Cooke et al. (1995) were the first to report that patients with RTT have thyroid dysfunction, which displays a significant decrease in serum total T4 concentration compared to the reference range. Stagi et al. (2015) also reported abnormal thyroid function in RTT, showing that serum T4 levels are elevated in patients with RTT. These two studies have shown contradictory results regarding the serum T4 levels. We cannot describe whether the discrepancies depend on the blood sample or the technique, and these phenomena are of interest for the study of thyroid function on RTT. Future case reports are needed to better interpret the alterations in serum T4 levels with RTT. In contrast, the dysgenesis of neurite length in MeCP2-deficient cells was significantly restored by the administration of IGF-1, whereas IGF-1 concentration in culture media was enhanced by the administration of T3 (de Souza et al., 2017). Therefore, the neuropathological findings observed in RRTs may be due to a decrease in IGF-1 levels, and thyroid hormones may have an indirect effect. Furthermore, MeCP2-knockout cells show that thyroid hormone-related genes, such as hormone transporters and deiodinases, are altered in these cells as compared to normal cells (de Souza et al., 2019). Therefore, MeCP2 probably has a significant influence on the assembly of the thyroid system in the body. In contrast, experimental hypothyroidism leads to alterations in MeCP2 expression in the cortex and liver of rodents. Uchida et al. (2021) reported that hypothyroid pups indicated a decrease in MeCP2 staining signals in cortical layers II–IV; however, the expression of MeCP2 mRNA was not altered. In contrast, Bunker et al. (2017) showed that neonatal exposure to antithyroid agents led to a decrease in MeCP2 mRNA expression in the liver; however, translated products did not change. Although there is not a clear explanation regarding the behavior of MeCP2 transcripts and translated products differs between organs, these results suggest that thyroid hormones have a significant effect on the expression of MeCP2, at least, and the relationship between thyroid function and MeCP2 might be that of reciprocal interactions rather than one-way interactions (Table 4). Further studies are needed to clarify the molecular mechanisms by which thyroid hormones affect MeCP2 expression.

**TABLE 4:** Thyroid function disorders and RTT.

Species	State	References	Relationship
Human	RTT	Cooke et al. (1995)	T4 ↓
Human	RTT	Stagi et al. (2015)	T4/T3 ↑
Cell	MeCP2-knockout cells	de Souza et al. (2017)	IGF-1 ↓
Cell	MeCP2-knockout cells	de Souza et al. (2019)	Mct8 ↓
Mouse	Experimental hypothyroidism (MMI/perchlorate)	Uchida et al. (2021)	MeCP2 protein ↓
Mouse	Experimental hypothyroidism (PTU)	Bunker et al. (2017)	MeCP2 mRNA ↓

*RTT, Rett syndrome; MMI, methimazole; PTU, propylthiouracil.*

Fukuda et al. (2005) reported delayed corticogenesis in MeCP2-deficient mice. Interestingly, the cortex of MeCP2-deficient mice has no parvalbumin neurons at postnatal day 14, and its expression catches up at 6 weeks after birth (Fukuda et al., 2005). Parvalbumin-expressing neurons contribute to aspects of the RTT phenotype; genetically modified mice specifically defecting MeCP2 on parvalbumin neurons indicate distinct RTT-like phenotypes (Ito-Ishida et al., 2015). Hence, the functional defect of parvalbumin might have a profound causal relationship with the development of psychiatric disorders. Of course, this behavior of parvalbumin closely resembles that of histological alterations in thyroid hormone deficiency during the perinatal period. In addition, the article also reports immature cortical formation in the somatosensory cortex of MeCP2-deficient mice, a phenomenon also observed in hypothyroid mice. The histological abnormalities observed in MeCP2-deficient and hypothyroid mice share many similarities, suggesting that there are downstream overlapping molecular mechanisms. Further exploration of the common denominator with hypothyroidism may reveal the molecular mechanism of developmental delay in RTT.

## Conclusion

In this review, we discussed the effects of thyroid hormones on the neural architecture of the brain, and then concisely mentioned their relationship with parvalbumin and/or MeCP2. The role of thyroid hormones in brain development has been studied since the early 20th century, and many studies have been published accordingly. Most of them are based on histomorphological evaluation by Nissl and Golgi staining, and the products of these histological studies have had a significant impact on the directionality of the current research. Furthermore, in recent years, researchers have been able to develop genetically modified mice with knockdown of target genes, and the importance of the thyroid system in brain development is becoming clearer. Nevertheless, just as the elementary processes of memory have been discovered, the mechanism of memory has not yet been elucidated, and the mechanism of developmental delay caused by congenital hypothyroidism has not been clarified. However, through animal experiments and clinical studies, a decrease in parvalbumin neurons has been reproducibly observed in hypothyroidism, and this phenomenon has also been observed in the postmortem brains of patients with schizophrenia and autism. Such comparative analysis with other psychiatric disorders may provide clues to the pathogenesis of developmental delay. In addition, since abnormal thyroid function is observed in patients with RTT, the onset of diseases associated with developmental disorders may have a common molecular mechanism. On the other hand, even though mild perinatal thyroid hormone deficiencies or even early treatment with levothyroxine, children who experience thyroid hormone deficiency during the perinatal period still retain the signatures of temporary hormonal defects in the adult brain. This case strongly indicates that thyroid hormones are essential for brain development. Hence, the study of congenital hypothyroidism and brain development remains a fascinating research topic.

## Author Contributions

KU wrote the manuscript. KU and MS collected the relevant research manuscript for the review. Both authors contributed to the article and approved the submitted version.

## Conflict of Interest

The authors declare that the research was conducted in the absence of any commercial or financial relationships that could be construed as a potential conflict of interest.

## Publisher’s Note

All claims expressed in this article are solely those of the authors and do not necessarily represent those of their affiliated organizations, or those of the publisher, the editors and the reviewers. Any product that may be evaluated in this article, or claim that may be made by its manufacturer, is not guaranteed or endorsed by the publisher.
